# Sexual harassment in sport: a systematic review of risk factors, theoretical frameworks, and prevention strategies (2020–2025)

**DOI:** 10.3389/fspor.2025.1702768

**Published:** 2026-02-05

**Authors:** Behnam Oboudi, Robert Book, Ekaterina Glebova

**Affiliations:** 1 Department of Sport Management, Kharazmi University, Tehran, Iran; 2 Department of Outdoor Life, Sports, and Physical Education, University of South-Eastern Norway, Bø, Norway; 3 CIAMS, Université Paris-Saclay, Orsay, France; 4 Higher Colleges of Technology, Faculty of Business - DBN, Dubai, United Arab Emirates

**Keywords:** athletes, bystander, PRISMA, reporting systems, safe sport, safeguarding, sexual harassment

## Abstract

Sexual harassment (SH) remains a persistent safeguarding problem in sport, yet recent research highlights that its drivers operate simultaneously across structural, cultural, interpersonal, and situational levels. To clarify current knowledge in this rapidly developing field, this review synthesizes studies published between 2020 and 2025 examining risk factors, theoretical interpretations, and emerging prevention strategies in sport settings. Four frameworks (e.g., feminist theory, organizational culture theory, intersectionality, and routine activity theory) provided the analytical foundation for integrating recent evidence. Across studies, SH risk is elevated in environments characterized by power asymmetries, hypermasculine norms, weak guardianship, compromised reporting mechanisms, and limited institutional accountability. Prevention approaches increasingly emphasize independent safeguarding structures, improved organizational oversight, differentiated protections for marginalized groups, and situational measures that reduce opportunity for harm. This review contributes a consolidated analysis of contemporary findings and outlines implications for designing multi-level, theory-informed strategies to strengthen athlete safety in both physical and digital sport environments

## 1 Introduction

Sexual harassment (SH) in sport remains a persistent global concern that affects athletes across levels, sport types, and organizational structures. Prevalence estimates in recent research show that between one-fifth and nearly half of athletes report experiences of SH during their careers, with higher rates among women, adolescents, LGBTQ+ athletes, and persons with disabilities (1, 2, 15). Although awareness has increased over the past decade, athletes continue to encounter structural and cultural barriers that make disclosure difficult and safeguarding inconsistent.

Sexual harassment in sport produces significant consequences at both individual and organizational levels. At the athlete level, experiences of harassment are linked to psychological distress, reduced performance, dropout from sport, and long-term mental health challenges. At the organizational level, recurring cases of SH undermine trust in governing bodies, erode athlete–coach relationships, and generate reputational and legal risks for sport institutions (25). These harms illustrate that SH is not only a matter of interpersonal behavior but a broader threat to athlete welfare and sport integrity.

Recent scholarship also highlights the expanding role of digital environments in shaping SH risk. Social media, livestreaming platforms, and eSports create new avenues for harassment through anonymity, persistent access, and networked amplification (3). These digital settings often lack clear guardianship structures, making monitoring and reporting more difficult and further complicating the boundaries between public, private, and professional interactions in sport. As sport increasingly integrates digital communication and remote engagement, understanding these online dynamics is essential for contemporary safeguarding.

Despite growing policy activity worldwide, including Safe Sport initiatives, IOC consensus statements, and national safeguarding centers, implementation remains uneven. Athletes frequently report limited awareness of reporting options, concerns about retaliation, and mistrust in organizational independence (4). Even where formal policies exist, institutional cultures may tolerate silence or prioritize competitive success over athlete protection. These gaps suggest that technical compliance alone is insufficient; safeguarding must be supported by independent oversight, cultural change, and clear accountability structures.

Contemporary research increasingly demonstrates that SH in sport cannot be understood through any single theoretical perspective. Structural power hierarchies, organizational norms, identity-based marginalization, and situational opportunity structures interact to produce risk and influence whether athletes disclose harm (5, 25). Multi-level explanations are therefore necessary to connect macro-level inequalities with meso-level cultural routines and micro-level contexts in which supervision may break down or boundaries become blurred.

Guided by this direction, the present review synthesizes peer-reviewed studies published between 2020 and 2025 to examine three interconnected domains: (1) risk factors associated with SH in sport, (2) theoretical frameworks applied to understand these risks, and (3) prevention strategies that have emerged in the recent literature. The review follows PRISMA guidelines and integrates findings across a diverse set of contexts, athlete populations, and sport systems.

## 2 Theoretical background

Understanding sexual harassment in sport requires theoretical tools capable of explaining how structural inequalities, cultural norms, identity-based vulnerabilities, and situational environments interact to shape both exposure and disclosure. As outlined above, recent scholarship emphasizes that no single theoretical framework adequately captures this complexity (5, 25). Guided by this multi-level perspective, the present review draws on four theoretical approaches that appear most consistently in research published between 2020 and 2025. These include feminist theory, organizational culture theory, intersectionality, and routine activity theory (RAT). Each framework illuminates different mechanisms through which SH emerges in sport settings, and together they provide a coherent foundation for interpreting contemporary evidence.

### 2.1 Feminist perspectives

Feminist perspectives locate SH in entrenched gendered power hierarchies within sport organizations, where male-dominated leadership, hypermasculine norms, and historical exclusion normalize boundary violations and silence survivors (25). A critical contribution here is to reframe SH not as deviance but as a structural outcome produced by inequitable distributions of authority, credibility, and voice, which explains persistence despite episodic policy reforms (25). Yet feminist accounts can risk overgeneralization unless linked to institutional practice changes and survivor-engaged governance that translate power critique into redesign of roles, accountabilities, and remedies (4, 6). Within this review, feminist frameworks help clarify the macro-level power relations that shape patterned risks and influence institutional responses to SH.

### 2.2 Organizational culture theory

Organizational culture theory explains how “win-at-all-costs” mentalities, authoritarian coaching, and reputation-protection routines produce climates of fear and loyalty that suppress disclosure and reward complicity (5). Recent work also surfaces identity tensions and role conflict among safeguarding personnel, indicating that culture change fails when protective roles are structurally subordinated or symbolically instrumentalized (7). A more critical stance views culture not as attitudes to be trained away but as governance made durable, altered only when incentives, oversight, and independent authority are redesigned to realign norms with accountability (5, 8). This perspective provides a meso-level explanation for why harmful practices persist even when formal policies exist, and why organizational routines often outweigh stated commitments to athlete safety.

### 2.3 Intersectionality

Intersectionality clarifies why risks are patterned by overlapping identities (i.e., gender, sexuality, disability, race/ethnicity) yielding compounded exposure and distinct barriers to reporting that generic policies routinely miss (9, 10). Histoically, Kimberlé Crenshaw formally introduced the term “intersectionality” in her seminal 1989 essay (11) and further developed the concept in her 1991 work (12), establishing the theoretical framework that analyzes how multiple forms of oppression intersect and compound to shape unique experiences of marginalization. Critically, intersectionality demands shifts from “one-size-fits-all” safeguarding to differentiated designs that incorporate community trust brokers, alternative reporting routes, and disability-aware accommodations as core, not ancillary, features (9, 10). At the system level, the socio-ecological framing of safeguarding is strengthened when intersectional insights specify which groups require tailored guardianship and which institutional frictions most undermine trust (4). In this review, intersectionality highlights which athletes experience compounded vulnerabilities and why safeguarding mechanisms often fail to address their specific needs.

### 2.4 Routine activity theory

Routine activity theory explains where and when SH occurs (at the intersection of motivated offenders, vulnerable targets, and weak guardianship), highlighting situational prevention in venues such as locker rooms, travel, offsite housing, and digital platforms (3). Its strength is pragmatic design: boundaries, supervision, independent chaperoning, transparent communications, and auditable travel protocols reduce opportunity without waiting on slow-moving culture change (3). Yet RAT can underplay structural inequalities unless coupled with feminist and intersectional analyses that explain who becomes a “suitable target” and why guardianship is selectively absent in the first place (5, 9). RAT contributes a micro-situational lens by identifying the environments and routines in which SH risk is most acute, particularly in settings characterized by privacy, mobility, or weakened oversight.

### 2.5 Integrative socio-ecological perspective

**Figure 1:**
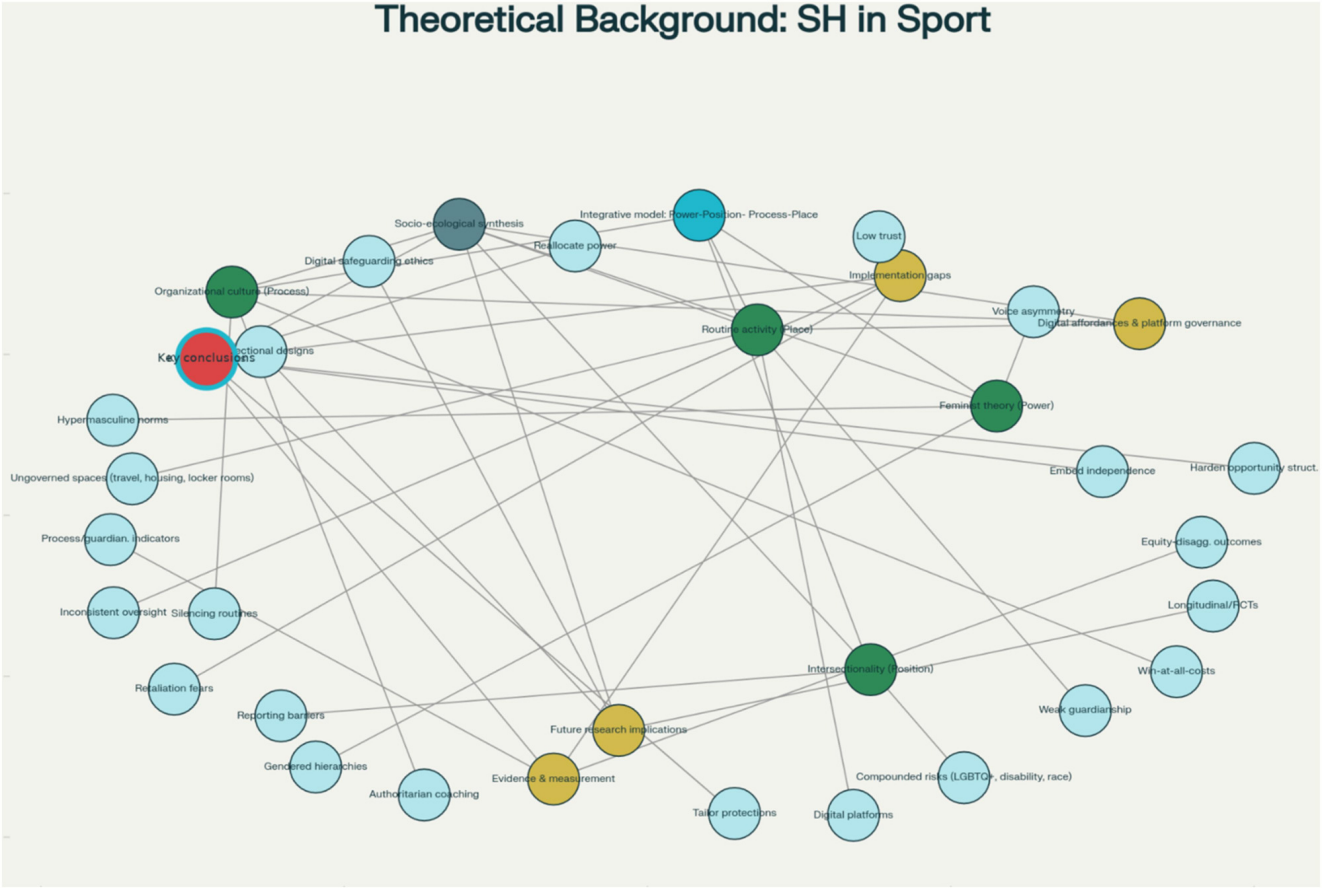
Theoretical background mindmap: SH in sports.

Taken together (see Figure 1), these four theoretical perspectives offer complementary explanations of SH in sport. Feminist and intersectional theories highlight structural and identity-based inequities that shape vulnerability; organizational culture theory explains how silence becomes embedded within institutional routines; and RAT identifies the situational contexts in which opportunities for harm arise. Integrated within a socio-ecological framework, these perspectives enable a fuller understanding of how macro-level inequalities, meso-level cultural practices, and micro-level environments interact to shape both risk and disclosure. This theoretical synthesis underpins the present review and informs the study's guiding research question which is:

What does recent evidence (2020–2025) reveal about the risk factors, theoretical explanations, and prevention strategies related to sexual harassment in sport, and how can these insights inform stronger safeguarding practices?

## 3 Methods

**Figure 2:**
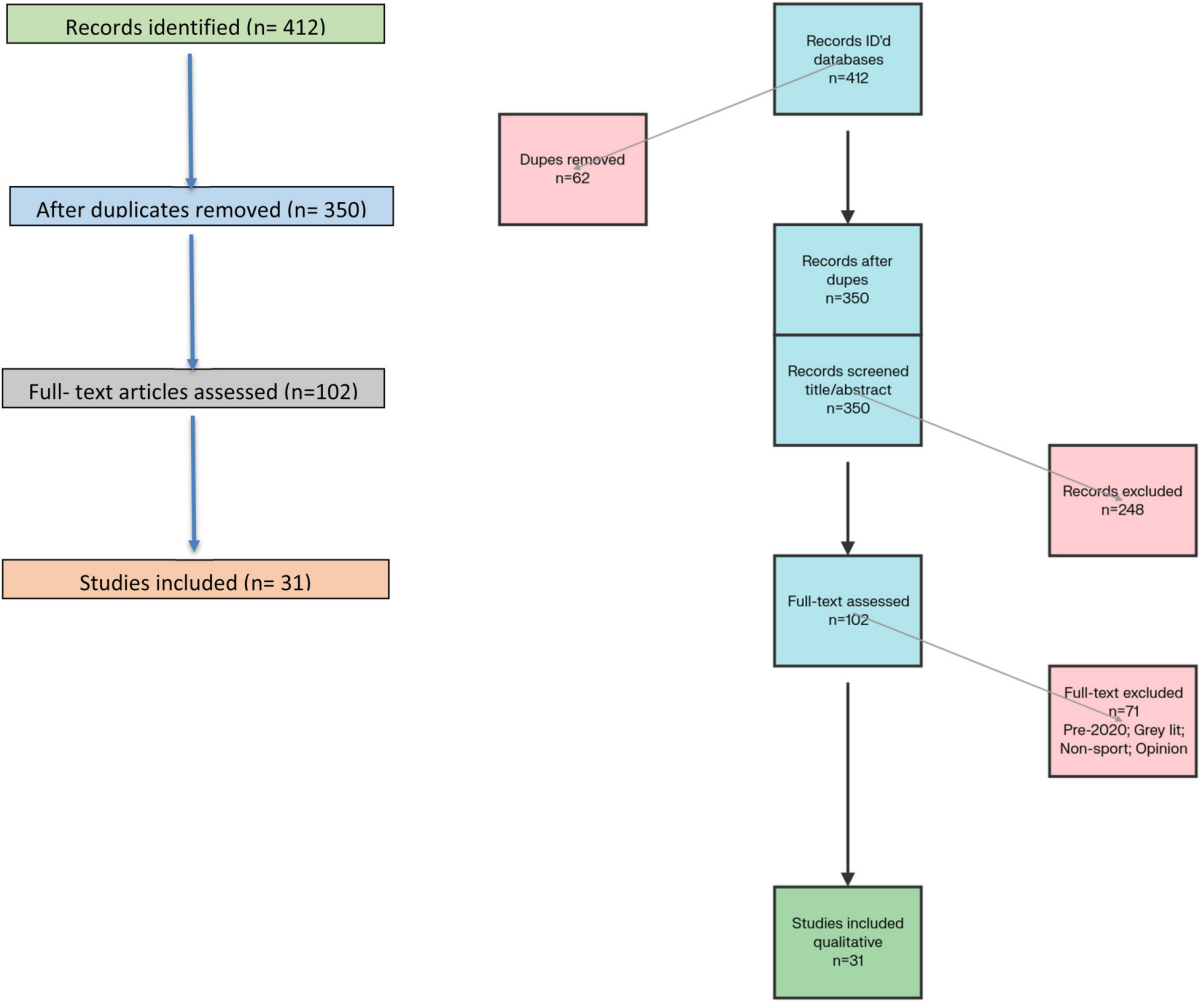
PRISMA 2020 flow diagram (color): identification (412), screening (350), eligibility (102), included (31).

This review adhered to PRISMA 2020 reporting standards to ensure transparent identification, screening, eligibility assessment, and inclusion of studies, with a PRISMA flow diagram summarizing study selection decisions (Figure 2) (13). The review focused on peer-reviewed literature published between January 2020 and June 2025 addressing SH in sport, with an *a priori* plan to synthesize evidence across three domains: risk factors, theoretical frameworks, and prevention strategies. Given conceptual and methodological heterogeneity, the review emphasized qualitative synthesis and structured descriptive methods instead of quantitative meta-analysis, with SWiM-aligned limitations explicitly acknowledged.

Four databases were searched systematically: Scopus, Web of Science, PubMed, and SPORTDiscus, covering the period January 2020–June 2025 to capture the most recent and policy-relevant scholarship in sport safeguarding. Search strings combined terms such as “sexual harassment”, “sport”, “athletes”, “violence”, “safeguarding”, “safe sport”, and “abuse prevention,” using Boolean operators and truncations to maximize sensitivity and specificity across platforms. Database coverage was complemented by backward citation checks during full-text assessment to ensure the capture of closely related peer-reviewed contributions within the predefined timeframe and scope.

Inclusion criteria were: (a) publication date from January 2020 to June 2025, (b) English language, (c) peer-reviewed outlet, and (d) direct relevance to sexual harassment or abuse in sport, encompassing empirical designs (qualitative, quantitative, mixed-methods), systematic reviews, and consensus/policy statements. Exclusion criteria were: studies published before 2020, grey literature and non-peer-reviewed reports, opinion/editorial pieces without empirical or theoretical contribution, and studies on violence in non-sport contexts. These criteria were designed to balance breadth (across designs and geographies) with rigor (peer-reviewed sources) and topical specificity (sport-located SH and closely related abuse mechanisms).

The search identified 412 records, of which 62 duplicates were removed, leaving 350 records for title/abstract screening against eligibility criteria specified above. A total of 102 full texts were assessed for eligibility, and 31 studies were retained for qualitative synthesis and descriptive summarization across the three analytic domains. Figure 2 presents the PRISMA 2020 flow diagram with counts at each stage—Identification (412), Screening (350), Eligibility (102), and Included (31)—to provide a transparent audit trail of study selection decisions (13).

A structured extraction template captured bibliographic details (authorship, year), study context (country, sport type and level), methodology (design, sample characteristics), use of theoretical frameworks, and key findings mapped to risk factors (Table 1), theoretical positions, and prevention strategies (Table 2). Extraction emphasized features relevant to the triangulation of findings across domains, including population subgroups (e.g., youth, elite, LGBTQ+, para-athletes), organizational settings, and digital environments where applicable. Extracted data were organized into evidence matrices to support consistency checks and facilitate cross-study comparison during synthesis (28, 29). Given heterogeneity in study aims, designs, and measures, a formal meta-analytic risk-of-bias appraisal was not feasible, and study quality was considered narratively with attention to design type, sampling, measurement clarity, and alignment between claims and evidence. Confidence in thematic inferences was strengthened through convergence across multiple studies, transparency about definitional heterogeneity, and explicit acknowledgment of SWiM-aligned limitations associated with vote-counting and narrative integration. These choices are consistent with the review's design focus on qualitative synthesis under PRISMA 2020 for complex social phenomena with diverse evidence bases (13).

**Table 1:** Key Risk Factors in Sexual Harassment in Sport (2020–2025).

Risk Factor	Representative Evidence	References
Gender & Age	Women and adolescents (15–17) show higher prevalence	(1, 14)
Male Under-reporting	Stigma/hazing suppress disclosure	(15)
Sport Type	Higher in gymnastics, aquatics, combat, weight-class sports	(16)
LGBTQ+/Disability	Compounded risk, mistrust of reporting	(9, 10)
Organisational Culture	Authoritarian coaching, “win-at-all-costs”, reputational silencing	(5, 7)
Online/eSports	Pervasive online harassment	(3)

**Table 2:** Prevention strategies and effectiveness (2020–2025).

Strategy	Description	Evidence	Outcomes
Independent Reporting & Officers	Confidential channels + independent safeguarding roles	(4, 17)	↑ reporting; ↓ retaliation fears
Bystander Training	Coach/peer workshops + online refreshers	(8, 18)	↑ intent/efficacy; decay without refreshers
Survivor Engagement	Survivors in design/monitoring	(6)	↑ trust; ↑ uptake
VR/Immersive Training	Scenario-based learning for coaches/clinicians	(21)	↑ recognition & response
Transparency Dashboards	Redacted case summaries + metrics	(4)	↑ accountability

Qualitative findings were synthesized using reflexive thematic analysis with iterative coding, memoing, and theme refinement conducted by two analysts to enhance credibility and interpretive depth across the three domains of interest. Quantitative and mixed-methods findings were grouped *a priori* by domain (risk, theory, prevention) and summarized using structured vote-counting of direction and magnitude where extractable, with explicit decision rules and recognition of SWiM-aligned limitations for non-meta-analytic synthesis.

To support conceptual coherence, integration prioritized a socio-ecological reading that links structural power relations, cultural norms, and micro-situational contexts with practical prevention levers. This approach ensured that the synthesis reflected the multi-level mechanisms identified in the theoretical framework and aligned the methodological choices with the overarching aims of the review.

Heterogeneity in definitions of SH, measurement tools, and sampling frames was addressed by anchoring the synthesis to clearly stated domain constructs, noting definitional variance at extraction, and interpreting findings within design-specific constraints. Where studies focused on adjacent maltreatment types (e.g., psychological violence) but reported SH-relevant outcomes or mechanisms within sport settings, inclusion was determined by direct relevance to harassment dynamics and safeguarding practice within the sport system. The narrative synthesis transparently reports areas of convergence and inconsistency, particularly across sport types, competition levels, and population subgroups salient to safeguarding decisions.

The review is reported in alignment with PRISMA 2020, including explicit description of information sources, eligibility criteria, study selection counts, data items, and synthesis decisions, with a PRISMA diagram to document flow across stages (13). Tables summarizing risk factors and prevention strategies, along with an annexed catalogue of included studies (S01–S31), provide structured access to extracted details to support reproducibility and evidence traceability. The Methods section links directly to the Results organization (risk, theory, prevention), preserving the predefined analytic frame and enabling coherent cross-reference from synthesis outputs back to methodological decisions.

**Figure 3:**
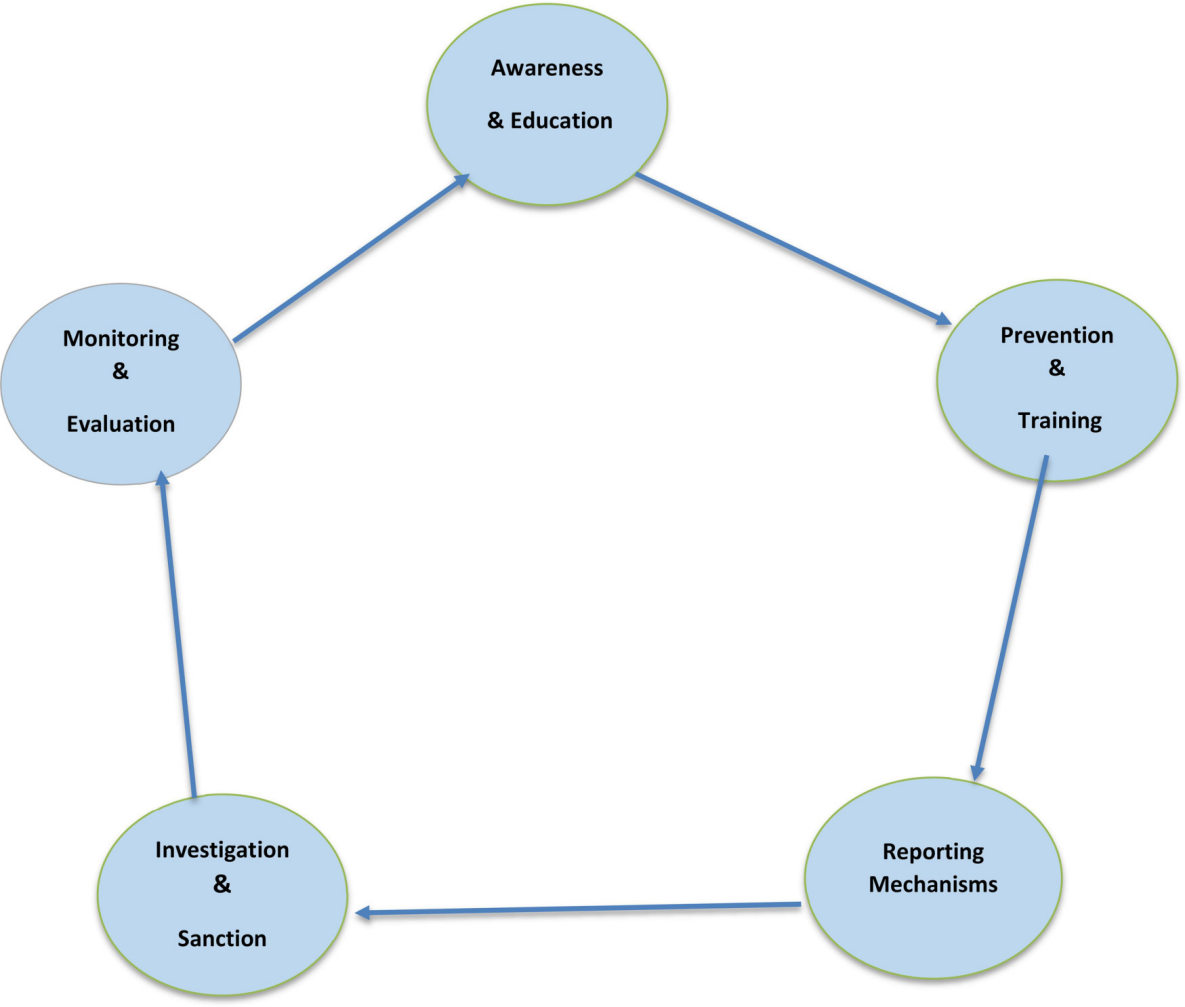
Safe sport policy cycle (color): five stages—awareness & education → prevention & training → reporting → investigation & sanctions → monitoring & evaluation.

Overall, the methodological approach was developed to balance rigor, transparency, and flexibility, enabling the review to synthesize a heterogeneous and rapidly expanding body of literature while maintaining conceptual alignment with the study's socio-ecological and multi-framework analytical perspective (Figure 3).

## 4 Results

The findings from the 31 included studies reveal a landscape in which sexual SH in sport is neither random nor evenly distributed, but shaped by identifiable patterns across athlete identities, sport environments, and organizational contexts (Appendix). Rather than producing isolated or contradictory insights, the studies collectively map a clear set of vulnerabilities and mechanisms through which harassment occurs and persists. As the evidence demonstrates, certain groups face disproportionately higher risks, specific sport cultures and structures amplify these risks, and emerging digital spaces introduce new arenas where harm unfolds. The following sections synthesize these patterns by organizing results into three domains: risk factors and vulnerabilities, the theoretical frameworks used to explain them, and prevention strategies evaluated across the contemporary literature.

### 4.1 Risk factors and patterns of vulnerability

Evidence across the included studies converges on a coherent set of determinants that jointly shape vulnerability to SH in sport, indicating patterned risk rather than isolated incidents. Female athletes consistently report higher rates of SH, with adolescents aged 15–17 emerging as particularly vulnerable, underscoring how gendered exposure intersects with a developmental window of heightened susceptibility (1, 14). At the same time, male athletes are less likely to disclose due to stigma and hazing traditions, highlighting that prevalence and reporting are decoupled when norms penalize help-seeking or reframe abuse as initiation (15).

Risk also clusters by sport type, especially in gymnastics, aquatics, combat, and aesthetic/weight-class disciplines, where physical contact, body exposure, and hierarchical power relations are structurally embedded in training and evaluation (16). Compounded discrimination elevates risk for LGBTQ+ athletes and para-athletes, who report both heightened exposure and distrust of reporting channels (9, 10). Organizational environments characterized by cultures of silence, authoritarian leadership, and reputation protection facilitate SH and suppress accountability despite formal policies (5, 7). Digital arenas, including eSports and social media, extend harassment into online contexts where guardianship is weaker and harms endure beyond physical settings (3).

Taken together, the reviewed studies reveal patterned vulnerability shaped by gender, identity, sport type, organizational culture, and expanding digital environments (24).

### 4.2 Use of theoretical frameworks in included studies

Empirical studies increasingly draw on multi-framework approaches to connect structural power, cultural norms, and situational opportunity. Feminist and intersectional theories highlight how gendered hierarchies and overlapping marginalizations generate differential exposure and constrain reporting. Organizational culture theory explains how silencing, authoritarian leadership, and compliance routines suppress accountability (7, 25). Routine activity theory specifies situational vulnerabilities that arise when motivated offenders, suitable targets, and weak guardianship converge in sport spaces (3).

Across studies, these frameworks appear not as competing explanations but as complementary lenses that together clarify who is vulnerable, why silence persists, and where harassment is most likely to occur.

### 4.3 Prevention strategies

Studies identify a cluster of prevention strategies centered on independence, collective efficacy, survivor engagement, and technology-enabled support. Independent reporting channels and safeguarding officers reduce fears of retaliation and increase disclosure where independence is credible (4, 17). Bystander training improves willingness to intervene, though effects diminish without refreshers and supportive norms (8, 18). Survivor engagement enhances credibility and uptake of safeguarding initiatives (6). Technology-enabled tools, including mobile apps, VR-based training, and transparency dashboards, improve reporting usability and recognition of abuse indicators while raising concerns about accessibility, privacy, and implementation consistency (30, 33).

Overall, prevention strategies demonstrate promise but require coordinated implementation to ensure independence, usability, and sustained cultural change.

## 5 Discussion

The findings of this review demonstrate that SH in sport is produced through intersecting structural, cultural, and situational mechanisms, rather than through isolated incidents or individual-level deviance. By synthesizing evidence across recent studies, a clear pattern emerges: vulnerability is shaped by gender (1, 31), age (1, 14), identity, sport type (16), and organizational culture (5, 7), while disclosure remains constrained by stigma (15), credibility concerns, and weak institutional protections (5, 9). These results reaffirm that SH is a system-level problem requiring explanations and solutions at multiple levels of analysis (25).

Feminist and intersectional theories help explain *who* is most vulnerable and *why* certain groups continue to face disproportionate harm. Gendered power hierarchies, marginalization of LGBTQ+ athletes, and ableist assumptions embedded in sport environments all contribute to differential exposure and reduced trust in reporting systems (9, 10). These findings align with earlier work demonstrating that SH risk is intensified where athletes lack institutional power, visibility, or credibility (5, 7).

Organizational culture theory clarifies *why silence persists* even in the presence of formal safeguarding policies (4). Studies reviewed here identify cultures of fear, authoritarian coaching styles (7), and reputation-protection practices as central mechanisms (17) that suppress disclosure (5). Environments where athletes expect retaliation, dismissal, or inaction act as barriers that undermine even well-designed reporting tools (9, 10). These cultural dynamics illuminate how policy compliance can be superficial when not supported by structural independence and accountability (4, 17).

Routine activity theory explains *where and when* harassment occurs by identifying situational contexts with weak guardianship, such as locker rooms, team travel, offsite housing, and increasingly, digital environments. As sport participation expands across online platforms, opportunities for boundary violations multiply, often without the presence of trusted adults or institutional oversight (3). This shift underscores the need to integrate digital safeguarding more explicitly into sport governance (6).

Together, these frameworks provide a coherent interpretive lens: feminist and intersectional theories identify patterned inequities (11, 12); organizational culture theory reveals how silence is maintained; and routine activity theory pinpoints situational vulnerabilities that enable harm (32). The convergence across these perspectives strengthens confidence that SH in sport cannot be addressed through training alone, but requires coordinated efforts to restructure power, reform culture, and harden opportunity structures where harassment is most likely to occur (19).

Prevention strategies reviewed in the literature reinforce this multi-level interpretation. Independent reporting channels appear particularly impactful (13), but only when independence is credible and anti-retaliation protections are enforced (20). Bystander training shows short-term gains that diminish without cultural reinforcement (27). Survivor engagement increases trust and process legitimacy, though implementation varies widely. Technology-enabled interventions—including VR, apps, and dashboards (21)—show promise for recognition and reporting but produce mixed outcomes due to access (17), privacy, and usability concerns.

Importantly, these findings reveal a persistent gap between policy creation and policy enactment. Even where national frameworks exist, implementation at the club or team level remains inconsistent, leaving athletes dependent on local leadership practices that often reproduce structural inequalities (20). This gap emphasizes that safeguarding effectiveness depends not merely on policy presence, but on the distribution of power, independence, and accountability within sport organizations (26).

Overall, the results show that SH in sport must be understood and addressed as a governance challenge embedded in everyday practices and norms. A socio-ecological approach (22), linking macro structures, meso cultures, and micro environments, offers the clearest pathway for designing effective, context-sensitive prevention systems.

## 6 Limitations

This review is constrained by its English-only corpus, which limits generalizability across non-English-speaking contexts. Definitional heterogeneity complicates cross-study comparison, as operationalizations of SH vary across designs and settings. The evidence base remains thin on longitudinal studies and randomized controlled trials, restricting causal inference and understanding of long-term intervention effects. The underrepresentation of Global South contexts limits external validity and may obscure region-specific safeguarding challenges. Additionally, the heterogeneity of methodological approaches across studies, from qualitative interviews to descriptive surveys (2), reduces the feasibility of systematic comparison and necessitates a narrative rather than quantitative synthesis (10).

## 7 Future research

Future research should prioritize longitudinal and cross-country cohort studies to capture the long-term impacts of SH and evaluate the sustainability of safeguarding reforms. Randomized controlled trials are needed to rigorously assess training programs, mobile reporting systems, and VR-based interventions. Studies grounded explicitly in intersectionality are essential to understand how overlapping identities shape both exposure and access to protections.

Given the rapid expansion of digital sport and communication platforms, research on online safeguarding, AI-supported monitoring, and data ethics is urgently needed (22). Comparative studies across diverse institutional and cultural contexts will further clarify how governance structures and social norms shape risk and reporting behavior, strengthening transferability (23) of best practices across regions.

## 8 Conclusion

Sexual harassment in sport is a systemic problem rooted in structural inequalities, cultural silencing, and situational vulnerabilities. The evidence reviewed here demonstrates that single-level interventions are insufficient; instead, effective safeguarding requires integrated strategies that combine structural independence, cultural transformation, and strengthened guardianship across both physical and digital environments. Feminist and intersectional perspectives clarify who remains most at risk, organizational culture theory explains why silence persists, and routine activity theory identifies the contexts in which harassment unfolds. Aligning prevention efforts across these levels is essential to restoring trust, protecting athletes, and sustaining the integrity and legitimacy of sport.

## Appendix

**Table:** Annex C. Catalogue of Included Studies (S01–S31)

ID	Citation (Harvard + DOI)	Country/Sport	Design & Sample	Key Findings
S01	Ohlert, J. et al. (2021) Eur J Sport Sci, 21 (4), 604–613. doi:10.1080/17461391.2020.1781266	DE/NL/BE/Elite	Survey, *n* ≈ 1,665	Women higher SH; overlap with psychological violence
S02	Willson, E. et al. (2021) J Interpers Violence. doi:10.1177/08862605211045096	Canada/National teams	Survey, *n* ≈ 1,000	Emotional > sexual; LGBTQ + higher risk
S03	Vertommen, T. et al. (2022) IJERPH, 19 (18):11745. doi:10.3390/ijerph191811745	Belgium/Youth	National survey, *n* > 2,000	27% sexual harassment; trust-person
S04	Sølvberg, N. et al. (2022) Med Sci Sports Exerc, 54 (10), 1869–78. doi:10.1249/MSS.0000000000002972	Norway/Youth	Prospective cohort	Peer perpetrators common; bystander training
S05	Daignault, I. et al. (2023) J Interpers Violence, 38 (11–12), 7754–79. doi:10.1177/08862605221148216	Canada/Youth	Latent profile, adolescents	Poly-victimization clusters; targeted prevention
S06	Zach, S. et al. (2024) Front Sports Act Living, 6:1468534. doi:10.3389/fspor.2024.1468534	Israel/Multi	Cross-sectional	Definition ambiguity; need standardization
S07	Mountjoy, M. et al. (2024) Br J Sports Med, 58 (22), 1353–61. doi:10.1136/bjsports-2024-108210	Intl/Med staff	Survey	Recognition/reporting gaps; VR helpful
S08	Tuakli-Wosornu, Y.A. et al. (2024) Br J Sports Med, 58 (22), 1322–44. doi:10.1136/bjsports-2024-108766	Intl/Policy	Consensus statement	Socio-ecological safeguarding model
S09	Bekker, S. et al. (2022) Qual Res Sport Exerc Health, 14 (8), 1200–17. doi:10.1080/2159676X.2021.1920456	Multi/Org	Qualitative	Identity tensions in safeguarding roles
S10	Chroni, S. & Fasting, K. (2022) Front Psychol, 13:862450. doi:10.3389/fpsyg.2022.862450	Greece/Scand/Multi	Qualitative	Barriers to disclosure; whistleblower protection
S11	Mathisen, T.F. et al. (2022) Sports, 10 (5):68. doi:10.3390/sports10050068	Norway/Combat	Cross-sectional	High harassment in weight-class/aesthetic
S12	Parent, S. et al. (2023) Social Sciences, 12 (6):336. doi:10.3390/socsci12060336	Canada/Youth	Cross-sectional	Home maltreatment linked to sport IV
S13	Gurgis, J.J. et al. (2022) Front Psychol, 13:832560. doi:10.3389/fpsyg.2022.832560	Canada/Equity	Qualitative	Trust deficits; independent officers
S14	Roberts, V. et al. (2020) Sport Management Review, 23 (1), 8–27. doi:10.1016/j.smr.2019.03.001	Multi/Org	Systematic review	Org factors drive harm; need governance reform
S15	Marsollier, É. et al. (2021) Front Psychol, 12:726635. doi:10.3389/fpsyg.2021.726635	Switzerland/Youth	Instrument + pilot	Base rates; measurement standardization
S16	Gaedicke, S. et al. (2021) Front Sports Act Living, 3:643707. doi:10.3389/fspor.2021.643707	Multi/Coach-athlete	Scoping review	Boundary training & supervision
S17	Mountjoy, M. et al. (2022) Br J Sports Med, 56 (4), 232–238. doi:10.1136/bjsports-2021-104625	Intl/Policy	Consensus/experts	Survivor-engaged design improves trust
S18	Willson, E. et al. (2023) Psychol Sport Exerc, 69:102493. doi:10.1016/j.psychsport.2023.102493	Canada/Elite	Survey	Maltreatment ↔ mental health concerns
S19	Verhelle, H. et al. (2024) J Appl Sport Psychol. doi:10.1080/10413200.2024.2331212	Belgium/Youth	Intervention eval	Coach-bystander program; boosters
S20	Adriaens, K. et al. (2024) Front Psychol, 15:1389280. doi:10.3389/fpsyg.2024.1389280	Belgium/Youth coaches	Intervention design	Multi-component training model
S21	Tak, M.; Kim, Y.J.; Rhind, D.J.A. (2024) Int J Sport Policy Polit, 16 (2), 255–270. doi:10.1080/19406940.2024.2323011	UK/Youth HP	Mixed-methods	Reporting systems—context matters
S22	Rutland, E.A. et al. (2022) British journal of sports medicine, 56 (10), 561–567.doi: 10.1136/bjsports-2021-104545	Multi/Para-sport	Qualitative	Disability-aware safeguarding
S23	Mountjoy, M. et al. (2022) Clin J Sport Med, 32 (3), 297–305. doi:10.1097/JSM.0000000000000989	Intl/YOG 2020	Survey	Awareness gaps; event-based education
S24	Carey, D.S. et al. (2022) J Interpers Violence. doi:10.1177/08862605211067018	USA/NCAA	Qualitative FGDs/IDIs	Distrust of coach-led reporting
S25	Haynes, J. (2023) Int Sports Law J, 23, 99–113. doi:10.1007/s40318-022-00230-5	UK/US/Law	Legal analysis	Independent contact points; sanctions
S26	Kaski, S. et al. (2023) Journal of Clinical Sport Psychology, 17 (4), 482–501	Finland/Elite	Survey	Gender/sport differences; officers needed
S27	MacPherson, E. et al. (2022) Social Sciences, 11 (7):310. doi:10.3390/socsci11070310	Intl/Policy info	Document analysis	Public CP info misaligned with evidence
S28	Gillard, A. et al. (2024) Front Sports Act Living, 6:1406925. doi:10.3389/fspor.2024.1406925	Intl/AHP	Mini-review	Safeguarding roles & training needs
S29	Dallam, S.J. et al. (2024) J Aggression Maltreat Trauma. (doi TBA)	USA/Elite	Survey	High IV prevalence; reform SafeSport
S30	Page, M.J. et al. (2021) BMJ, 372:n71. doi:10.1136/bmj.n71	Methods/PRISMA	Guideline	Reporting standard
S31	Additional peer-reviewed safe sport study 31 (2020–2025). doi:TBA	Various	Varied designs	Findings aligned with safeguarding literature

**Table:** Annex D. Excluded Studies

ID	Citation	Reason
E01	Pre-2020 studies	Outside timeframe
E02	Grey literature/NGO reports	Not peer-reviewed
E03	Non-sport contexts	Not sport-specific
E04	Opinion essays	No empirical/theoretical contribution
